# In vivo and in vitro activation of dormant primordial follicles by EGF treatment in mouse and human

**DOI:** 10.1002/ctm2.182

**Published:** 2020-09-27

**Authors:** Jiawei Zhang, Lei Yan, Yibo Wang, Shuo Zhang, Xueqiang Xu, Yanli Dai, Shidou Zhao, Zhen Li, Yan Zhang, Guoliang Xia, Yingying Qin, Hua Zhang

**Affiliations:** ^1^ State Key Laboratory of Agrobiotechnology, College of Biological Sciences China Agricultural University Beijing 100193 China; ^2^ Center for Reproductive Medicine Shandong University Jinan 250021 China; ^3^ State Key Laboratory of Plant Physiology and Biochemistry, College of Biological Sciences China Agricultural University Beijing 100193 China

**Keywords:** EGF, *in vivo* activation, noninvasive administration, premature ovarian insufficiency, primordial follicle activation

## Abstract

In the mammalian ovaries, dormant primordial follicles represent the reproductive reserve of individual females. Recently, stimulating the activation of primordial follicles in vitro has been practiced, making the utilization of those dormant follicles to treat female infertility possible. However, there are still lacks of effective upstream molecule and strategy to elevate follicle activation in vivo. In the current study, we revealed that growth factor EGF improved a transiently primordial follicle activation in mice by elevating the CDC42‐PI3K signaling activity, and EGF treatment also improved the activation and development of human follicles in ovarian cortical pieces. Using a liquid‐solid phase transition bio‐gel as a carrier, an efficient in vivo activation system was established by ovarian topical EGF administration to living mice. We found that EGF treatment led to an increase of primordial follicle activation in short time but had no effect on long‐term fertility in females. By establishing an inducible premature ovarian insufficiency (POI) mouse model through selectively ablating growing follicles in *Zp3‐Cre;iDTR* mice, we further revealed that our in vivo EGF treatment system improved primordial follicle activation and ovulation of POI ovaries significantly. Taken together, our results revealed that in situ ovarian EGF administration could improve the activation of primordial follicles in living animals, and manipulating activation and development of primordial follicles in vivo might be an efficient approach to improve reproductive health in women.

## Graphical abstract

Zhang et al. reveal that EGF is a physiological stimulator of dormant primordial follicle activation in both mouse and human. Using EGF as an activator, the current study established an efficient and **ovarian non‐invasive in vivo activation** system to improve the dormant follicle development in living animals. In this system, the ovarian surface is covered by the bio‐gel with EGF through a topical administration, then the dormant follicles on the cortical region are stimulated to active and develop. The experimental data shows that the system is efficient in both normal and primary ovarian insufficient females.

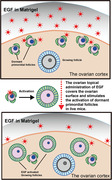

**Abbreviations**
ART: assisted reproductive technology
CDC42: cell division cycle 42
CL‐FOXO3a: cytoplasmic localization of FOXO3a
DT: diphtheria toxin
EGF: epidermal growth factor
ELISA: enzyme‐linked immunosorbent assay
FOXL2: forkhead box L2
FOXO3a: forkhead box O3
GCs: granulosa cells
GDF‐9: growth differentiation factor 9
iPOI: inducible premature ovarian insufficiency
IVA: in vitro activation
IVF: in vitro fertilization
mTORC1: mammalian target of rapamycin complex 1
PCOS: polycystic ovary syndrome
PD: postnatal day
PI3K: phosphatidylinositol 3 kinase
POI: premature ovarian insufficiency
preGC: pregranulosa cells
SCF: stem cell factor
SCID: severe combined immunodeficiency

## 1 INTRODUCTION

In mammalian ovaries, the oocyte is enclosed by somatic cells to form a follicle as the structural basis of female reproduction.^1^, ^2^ Although follicles are classified into different types in various stdies,^3^, ^4^ there are two major stages in their development, the dormant primordial follicles and the growing follicles.^5^ The current assisted reproductive technology, which mainly relies on the existence of growing antral follicles in the ovary, has been widely applied to treat female infertility in modern society. However, as the most abundant reproductive reserve, the understanding and utilization of dormant primordial follicles are still in its infancy.

In the ovary, most of primordial follicles are maintained at a quiescent state as the female reproductive reserve to maintain the long‐term reproductive lifespan of females. A small number of dormant primordial follicles are gradually recruited into the growing follicle pool through a process called primordial follicle activation, and finally contribute to female fertility.^6^ Although the understanding of the regulatory mechanisms in primordial follicle activation is still elusive compared to the well‐studied growing follicles, several important intrafollicle pathways in regulating this process have been revealed in recent decades.^2^, ^6^ The major signaling cascade in primordial follicles that controls the activation is preGC (pregranulosa cells) mTORC1‐oocyte PI3K cascade,^6^ and our recent study revealed that CDC42 in dormant oocytes is also an efficient and fast response target for activating primordial follicles.^7^ Moreover, the treatment of CDC42 activator dramatically improves the activation of dormant primordial follicles in cultured mouse ovaries.^7^ These studies have greatly increased our understanding of the inner mechanisms in controlling primordial follicle activation in mammals. With the research advances of primordial follicle activation, the application of activating dormant primordial follicles to overcome female infertility has been proposed. A novel concept in assisted reproductive technology called in vitro activation (IVA) has been applied in clinical practice, and several successful cases have been reported from different groups recently.^8^, ^9^, ^10^ However, the efficiency and convenience of IVA are not satisfactory,^11^ which block the wide application of this novel approach to treat infertility. Thus, the discovery of efficient targets or approaches to mimic the activating process under physiological conditions is essential to boost the application of this new concept.

The upstream regulatory mechanisms in controlling follicle activation under physiological conditions are still elusive, but several growth factors including stem cell factor (SCF),^12^, ^13^ growth differentiation factor 9 (GDF‐9),^14^, ^15^ and epidermal growth factor (EGF), etc., have been reported to act as local stimulators to promote follicle development. In those growth factors, EG plays as a multifunctional stimulator to improve the proliferation of granulosa cells,^16^, ^17^ the follicular viability,^18^ and the oocyte maturation.^19^, ^20^ Meanwhile, the expression of the EGF receptor has been reported to localize on follicles from primordial to antral stages of various mammalian species including humans,^16^, ^21^, ^22^, ^23^ showing that EGF is a general positive upstream regulator of follicle development in vivo. As paracrine or autocrine factors that act as upstream molecules to activate intracellular pathways, ovarian endogenous growth factors such as EGF might be an ideal candidate to mimic the physiological activation of primordial follicles in clinical treatment.

In the current study, we examined the role of several ovarian expressed growth factors in regulating the activation of dormant primordial follicles and tested the possibility of in vivo stimulating the activation of dormant primordial follicles with the growth factor in mice. Our results showed that EGF was the most efficient factor to transiently awaken the dormant primordial follicle in both mouse and human ovaries. Using a liquid‐solid phase transition bio‐gel as a carrier, our study showed that EGF was functional to improve the activation of dormant primordial follicles in vivo by an ovarian topical administration approach in both normal and inducible premature ovarian insufficiency (iPOI) mice. Although the in vivo EGF treatment had no marked effect on the fertility of normal female mice in long term, our results showed that this approach significantly increase the activation of dormant primordial follicles and the oocyte retrieval rate in inducible premature ovarian insufficiency (iPOI) mouse ovaries, which indicated a potent clinical strategy to improve the efficiency of assisted reproductive technology (ART) in POI patients.

## 2 RESULTS

### 2.1 EGF treatment stimulates the activation of primordial follicles in mouse ovaries

**FIGURE 1:**
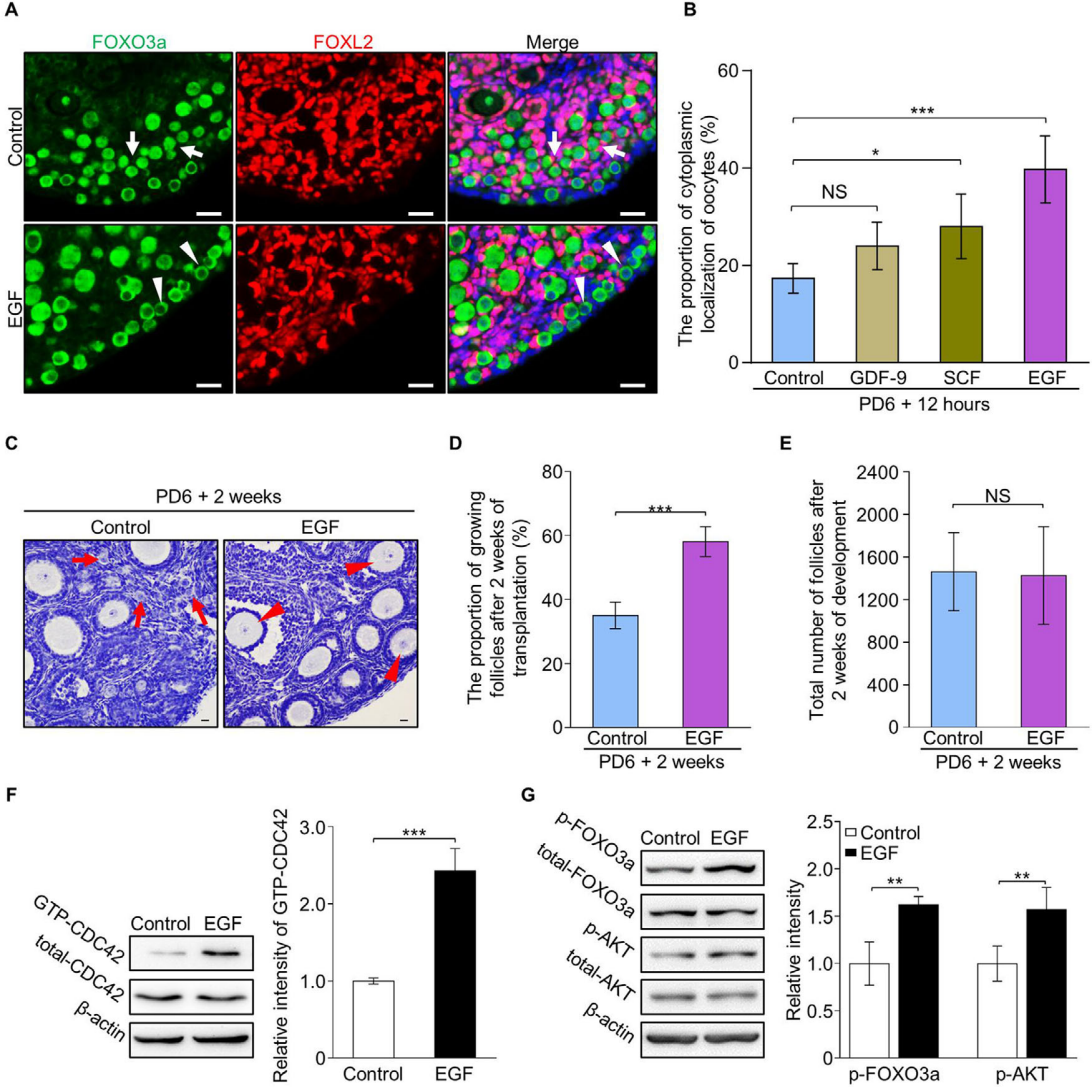
EGF treatment improves the activation of primordial follicles by elevating CDC42‐PI3K signaling in mouse ovaries. (A) FOXO3a (green) shuttled from the nuclei (arrows) to the cytoplasm (arrowheads) with oocyte activation. EGF treatment for 30 minutes markedly increased cytoplasmic localization of FOXO3a (arrowheads) in oocytes compared to controls. Granulosa cells were dyed with FOXL2 antibody (purple) to show the follicles and nuclei were dyed with a Hoechst counterstain (blue). (*n* = 6). (B) Quantification results revealed a significantly increased ratio of cytoplasm localization of FOXO3a in the oocytes of EGF (40 ± 6%), GDF‐9 (24 ± 4%) and SCF (28 ± 6%) treated ovaries compared to vehicle (17 ± 3%) treated ovaries (*n* > 3). (C) After 2 weeks of transplantation, primordial follicles in clusters (arrows) were detected in the control ovaries (*n* = 5) whereas an increased number of growing follicles (arrowheads) was observed in the cortical region of EGF‐treated ovaries (*n* = 5). (D) Follicle quantification showed a significantly increased percentage of growing follicles in the EGF group compared to controls (58 ± 4% vs 35 ± 4%) (*n* = 5). (E) EGF treatment had no effect on follicle survival (1428 ± 410 vs 1464 ± 372) (*n* = 5). (F) The CDC42‐GTP pull‐down assay showed that CDC42‐GTP levels were significantly increased in EGF‐treated ovaries compared to the controls. (G) Signaling studies in EGF‐treated and control ovaries, showing an enhanced level of p‐AKT and p‐FOXO3a in the EGF group compared to control ovaries. Levels of total FOXO3a, AKT, and β‐actin were used as internal controls. The experiments were repeated at least three times. In B, D, E, F, and G, data represent the mean ± SD of biological triplicate experiments. NS, *P *> .05, *P < .05, **P < .01, and ***P < .001, by two‐tailed unpaired Student's *t* test. Scale bars: 25 µm

Using a modified in vitro whole ovarian culture system established in our previous study,^7^ we aimed to screen an efficient stimulator to mimic the activation of primordial follicles under physiological conditions. Therefore, we first evaluated the activating effect of several growth factors that functioned in regulating follicle development, including GDF‐9, SCF, and EGF. Intact ovaries from postnatal day 6 (PD 6) females were cultured with or without different factors for 30 minutes, followed by being cultured in factor‐free medium for 12 hours, respectively. After culture, the expression of FOXO3a, which shuttled from the nucleus (Figure 1A, arrows) to the cytoplasm (Figure 1A, arrowheads) in oocytes during primordial follicle activation, was first detected. We found that the cytoplasmic localization of FOXO3a (CL‐FOXO3a) was slightly increased in GDF‐9 (Supplemental Figure S1A, arrowheads) and SCF (Supplemental Figure S1B, arrowheads) treated ovaries compared to the control, and a remarkable increase of CL‐FOXO3a oocytes was observed in the cortical region of EGF (100 ng/mL) treated ovaries (Figure 1A). Oocyte counting results confirmed that only a mild increase of CL‐FOXO3a oocytes was observed in GDF‐9 (24 ± 4%) and SCF (28 ± 6%) treated groups (Figure 1B), whereas a significantly increased ratio of CL‐FOXO3a oocytes was detected in EGF‐treated ovaries (40 ± 6%) compared to the ratio in the control (17 ± 3%) (Figure 1B). This result shows that EGF is an efficient growth factor to awaken the dormant primordial follicles.

To further examine the developmental capability of follicles after EGF treatment, the treated ovaries were allo‐transplanted under the kidney capsules of bilaterally ovariectomized adult mice. After 2 weeks of transplantation, a remarkable increase of growing follicles was found in EGF‐treated ovaries (Figure 1C, arrowheads). Follicle counting results confirmed that a major increase in the percentage of growing follicles in EGF‐treated ovaries compared to controls (58 ± 4% vs 35 ± 4%) (Figure 1D and Supplemental Figure S1C), indicating that EGF awakened dormant follicles developed normally in the ovary. Meanwhile, an identical number of total follicles in EGF‐treated and control ovaries (1428 ± 410 vs 1464 ± 372) (Figure 1E) showed that EGF treatment had no effect on follicular survival. Therefore, these results reveal that EGF is functional to improve the primordial follicle activation in mice.

### 2.2 EGF awakens the primordial follicles through CDC42‐PI3K signaling in mice

As a well‐studied endogenous growth factor, EGF has been reported to be a functional activator of CDC42,^24^ which is a highly efficient target for stimulating the activation of dormant primordial follicles.^7^ Therefore, we next investigated whether EGF stimulated the follicle activation through elevating CDC42‐PI3K signaling. Ovaries at PD 6 were cultured with or without EGF for 30 minutes, and a CDC42‐GTP pull‐down assay revealed that CDC42‐GTP levels were significantly increased in EGF‐treated ovaries compared to the vehicle control (Figure 1F). This result indicates that EGF treatment improves the activity of CDC42 in ovaries.

Since CDC42 stimulates the activation of dormant primordial follicle through elevating PI3K pathway,^7^ we next investigated whether EGF enhanced CDC42 signaling to increase activation of PI3K signaling in the ovary. Western blot analysis showed no change in total FOXO3a and AKT in EGF‐treated ovaries and controls, whereas the elevated phosphorylation of FOXO3a and AKT was found in the EGF group compared to the control group (Figure 1G), showing that EGF treatment led to the rapid activity of CDC42‐PI3K signaling in ovaries and therefore stimulated the activation of dormant primordial follicles in the ovary.

### 2.3 In vivo stimulating the activation of primordial follicles by a noninvasive ovarian topical administration of EGF in mice

**FIGURE 2:**
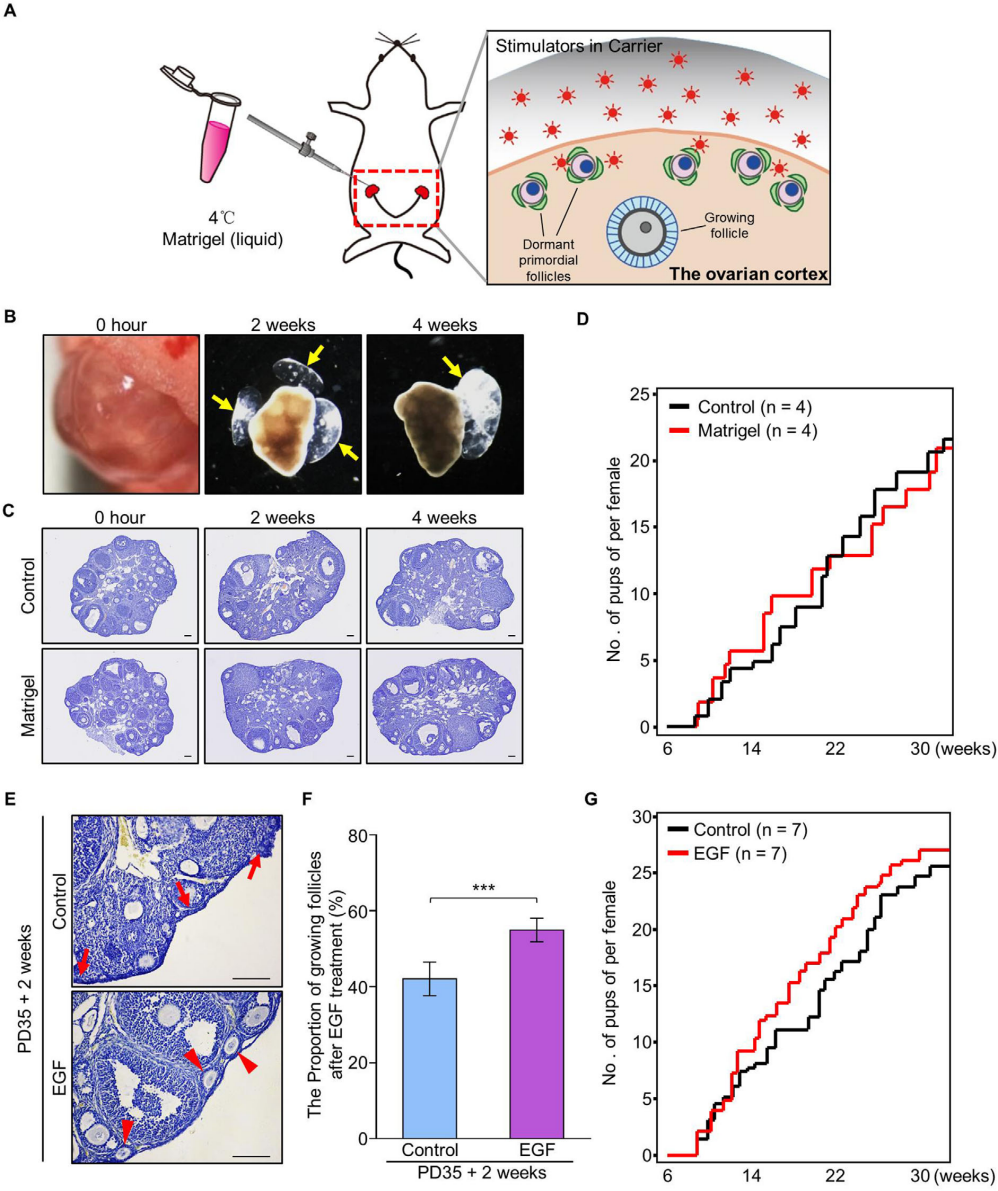
In vivo stimulation of the activation of primordial follicles by ovarian topical administration of EGF in PD35 mice. (A) Schema for the in vivo stimulator administration system to activate dormant primordial follicles in the ovaries of live mice. Using a liquid‐solid phase transition bio‐gel (Matrigel) as a carrier, the primordial follicle stimulator was delivered to the ovarian bursa and covered the ovarian surface in which the dormant primordial follicles localized. (B‐D) Validation of the system of ovarian topical Matrigel administration. (B) The Matrigel became solidification in ovarian bursa immediately after injection, and was existing on the ovarian surface at 2 and 4 weeks after injection (*n* = 4). (C) A normal follicular distribution was observed in the ovaries after 2 and 4 weeks of Matrigel treatment (*n* = 4) compared to control (*n* = 4). (D) Fertility check showed that Matrigel injection had no effect on female reproduction (*n* = 4). (E‐G) Ovarian topical administration of Matrigel‐carried EGF improved the proportion of growing follicles in the ovaries of adult mice. (E) Two weeks after in vivo EGF treatment, histological analysis showed that normal distribution of primordial follicles (arrows) was observed in the control ovaries (*n* = 3) whereas an increased number of growing follicles (arrowheads) was detected in the cortical region of EGF‐treated ovaries (*n* = 3). (F) Follicle counting revealed a significantly increased proportion of growing follicles in EGF‐treated ovaries compared to the proportion in controls (55 ± 3% vs 42 ± 4%) (*n* = 3). (G) EGF treatment had no dramatic effect on female fertility compared to that of the controls (*n* = 7). Data represent the mean ± SD of biological triplicate experiments. ***P < .001, by two‐tailed unpaired Student's *t* test. Scale bars: 100 µm

In the current IVA approach, the human ovarian cortical pieces were cut and treated with activators in the clinical practice, but the existence of primordial follicles was random and uncertain which decreased the efficiency of IVA greatly. All primordial follicles were localized on the cortical region of the ovary in vivo in mammals, we therefore attempted to establish an in vivo system to stimulate the activation of dormant primordial follicles to improve the efficiency of activation. As illustrated in Figure 2A, we hypothesized that enriching primordial follicle activators by a carrier on the ovarian surface might improve the activation of dormant primordial follicles in vivo with fewer side effects and tissue damage for animals.

We choose the growth factor reduced Matrigel, which is a phase transition bio‐gel in liquid at 4°C, and solidifies at body temperature (37‐39°C) as a carrier for injection,^25^, ^26^ and the gel was injected to the ovarian bursa which guaranteed the gel to keep in the ovarian surface. To validate the system, Matrigel was first injected into the ovarian bursas of adult females (PD35) and covered the ovarian surface without any damage on ovaries, as illustrated in Figure 2A and Supplemental Figure S2A. We found that the gel was existing on ovarian surface for more than 4 weeks (Figure 2B, arrows) after injection, and a normal ovarian development (Figure 2C) and fertility (Figure 2D) after treatment showed that gel injection had no effect on the general health of females. This result shows that the system of in situ injection of Matrigel is a safe approach, which could be used to carry the drug for in vivo follicle stimulation.

With the validated system, EGF (100 ng/mL) or vehicle was dissolved in liquid Matrigel and injected into the ovarian bursas to test the in vivo stimulating effect of EGF on primordial follicle activation. After 2 weeks of injection, although ovarian morphologies had no markedly difference between EGF‐treated and control ovaries (Figure 2E), follicle counting results revealed a significantly increased number of growing follicles in EGF‐treated ovaries compared to control (55 ± 3% vs 42 ± 4%) (Figure 2F), whereas an identical number of total follicles was observed (Supplemental Figure S2B). To confirm the in vivo stimulating effect of EGF, EGF (100 ng/mL) was also dissolved in hyaluronan and injected into the ovarian bursas of females at PD35. After 2 weeks of treatment, an increased proportion of growing follicles was observed in EGF‐treated ovaries compared to that of control (Supplemental Figure S3A‐3C), showing that EGF is functional to improve the activation of primordial follicles in vivo. To ensure the in vivo activating effect of EGF is from the topical administration, we performed intraperitoneal (i.p.) injection of EGF and tested the effect of follicle activation after treatment. In details, EGF with different dosages (identical dosage group: 100 ng/mL 20 µL or 5× dosage group: 500 ng/mL 20 µL) was i.p. injected to the females at PD35. After 2 weeks of treatment, we found that EGF with i.p. injection had no effect on the activation of primordial follicles in both histological analysis and follicle counting (Supplemental Figure S4A‐4C). Taking together, these results show that in situ EGF treatment increases the proportion of activated follicles in the ovaries of living animals.

We next investigated the long‐term effects of EGF treatment on the health and fertility of females. Normal increase of body weight (BW) (Supplemental Figure S2C) and visceral anatomy in animals indicated a normal development of females after 2 months (Supplemental Figure S2D) and 12 months (Supplemental Figure S2G) of ovarian topical EGF treatment. Histological analysis revealed a normal morphology of reproductive organs at 2 months (Supplemental Figure S2E) and 12 months (Supplemental Figure S2H) after treatment, confirmed that in vivo EGF treatment was a safe approach for stimulating follicle activation in our system. Interestingly, although an acute increase of primordial follicle activation was found after EGF treatment, fertility check results showed that EGF‐treated females gave birth normally till 30 weeks (Figure 2G). This was confirmed by an identical follicular distribution in EGF‐treated and control ovaries at 2 and 12 months (Supplemental Figure S2F and S2I) after treatment. Therefore, our results indicate that an acute increase in primordial follicle activation in vivo has no marked effect on the fertility of normal females, at least in mice.

### 2.4 Establishing an inducible POI mouse model by specific ablation of growing follicles in *Zp3‐Cre;iDTR* mice

**FIGURE 3:**
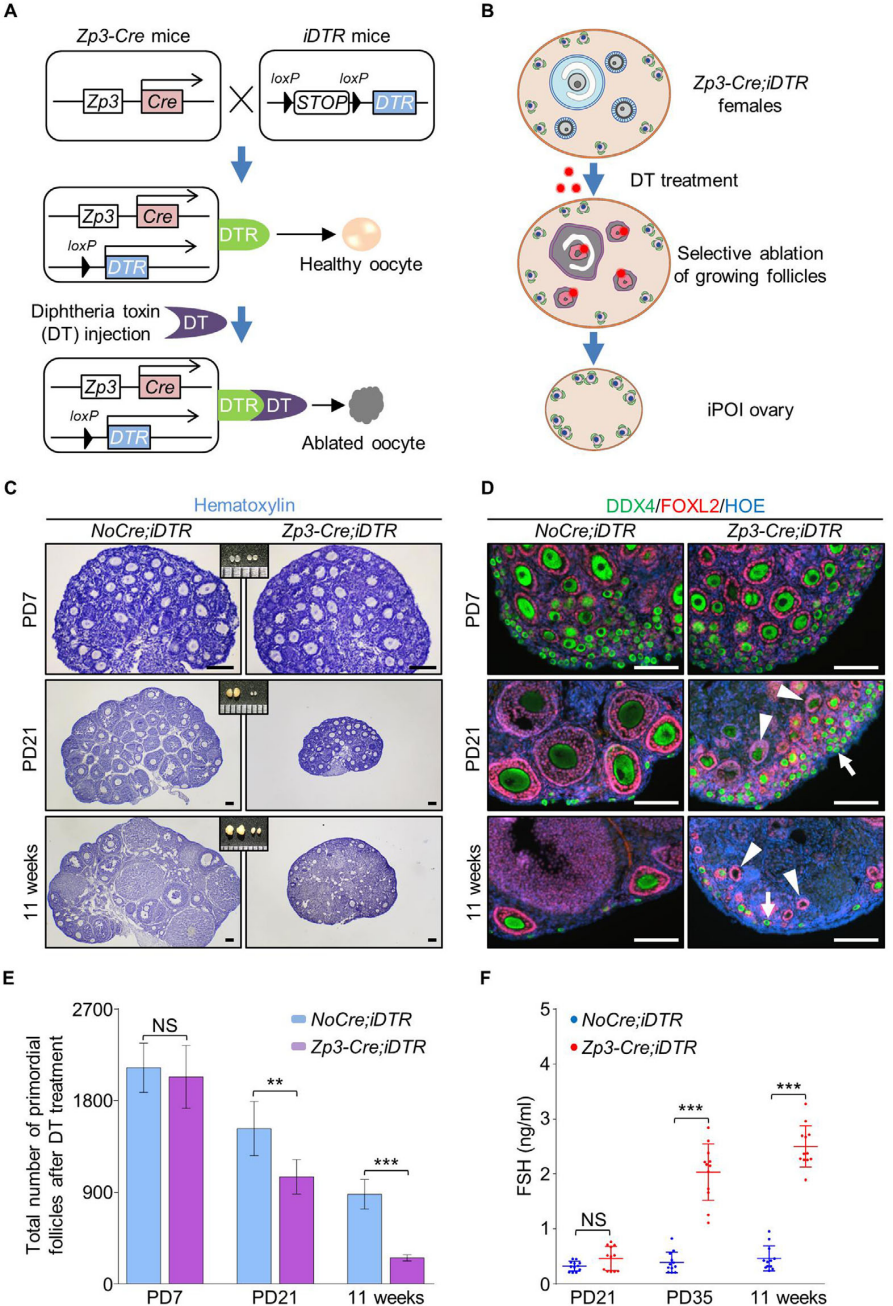
Establishing an inducible premature ovarian insufficiency mouse model by selective ablation of growing follicles in *Zp3‐Cre;iDTR* ovary. (A) Illustration of the inducible premature ovarian insufficiency (iPOI) model by DT‐induced ablation of growing follicles in *Zp3‐Cre;iDTR* ovaries. (B) Upon continuous DT administration, the growing follicles that contain *Zp3*‐expressing oocytes were selectively ablated to eliminate the follicle reserve in the ovary with no side effect on dormant primordial follicles and other ovarian cells. (C) DT was given to *Zp3‐Cre;iDTR* or *NoCre;iDTR* females for 10 consecutive weeks from 1 week after labor (10 µg/kg, one injection per week), and ovaries were examined at PD21 and 11‐weeks. Histological analysis showed a dramatic decrease in ovarian size with DT treatment in *Zp3‐Cre;iDTR* females. (D) Immunofluorescent labeling of follicles revealed that few growing follicles and a number of primordial follicles were found in the *Zp3‐Cre;iDTR* females after DT administration (green: DDX4, purple: FOXL2, blue: Hoechst, *n* = 3). (E) Primordial follicle counting showed a significantly decreased number of follicle reserve in ovaries of iPOI mice compared to the number in controls after DT treatment (*n* = 3 per group). (F) ELISA analysis showed that no change of serum FSH levels at 3 weeks old iPOI females after DT treatment (0.47 ± 0.21 vs 0.33 ± 0.09 ng/mL), but significantly elevated serum FSH levels were observed at PD35 (2.03 ± 0.49 vs 0.39 ± 0.18 ng/mL) and 11‐week old iPOI females (2.50 ± 0.36 vs 0.47 ± 0.22 ng/mL) compared to levels in controls (*n* = 12 per group). Data represent the mean ± SD of biological triplicate experiments. NS, *P *> .05, **P < .01 and ***P < .001, by two‐tailed unpaired Student's *t* test. Scale bars: 100 µm

Since EGF treatment stimulated the follicle activation but had no effect on fertility in wild‐type females, we next focused our study on whether the stimulating effect of EGF could improve the fertility of POI females. To mimic the ovarian symptoms in POI patients^27^ and avoid the side effects on nonfollicle cells and dormant primordial follicles, a mouse model in which the growing follicles were selectively ablated in the ovary was established by crossing *Zp3‐Cre* mice with Cre‐inducible diphtheria toxin receptor (*iDTR*) mice^28^ (Figure 3A). In these *Zp3‐Cre;iDTR* mice, the expression of the diphtheria toxin (DT) receptors (DTRs) is mediated by *Zp3‐Cre* in oocytes of growing follicles, thereby allowing the selective ablation of growing follicles upon administration of DT (Figure 3B) with no toxic effects for other cells and organs.^29^

To establish the model, DT (10 µg·kg^−1^ BW) was intraperitoneally injected into control *iDTR* or *Zp3‐Cre;iDTR* females once a week for 10 consecutive weeks from PD 7. In DT‐treated control *iDTR* females, a normal follicular distribution and ovarian development were observed during the treatment period from 1 to 11 weeks (Figure 3C and 3D, *NoCre;iDTR*), showing that oocyte development was not affected by DT treatment. In sharp contrast, the size of ovaries in treated *Zp3‐Cre;iDTR* mice markedly decreased after 2 weeks of DT treatment at PD 21 (Figure 3C). Immunofluorescent staining of oocytes (DDX4, green) and granulosa cells (FOXL2, red) showed that many growing follicles with irregular morphology and atresia oocytes (Figure 3D, arrowheads) were found in the DT‐treated *Zp3‐Cre;iDTR* ovaries at PD 21. After 10 weeks of DT treatment, although primordial follicles were still detected in the cortical region (Figure 3D, arrows) of *Zp3‐Cre;iDTR* ovaries, few healthy growing follicles (Figure 3C, 11 weeks) and many residual structures of follicles without oocytes (Figure 3D, arrowheads) were observed in the shrunken *Zp3‐Cre;iDTR* ovaries. These results showed an efficient and specific ablation of growing follicles in *Zp3‐Cre;iDTR* mice.

To confirm the phenotypes of POI in our model, the number of primordial follicle which represented the ovarian reserve of females was counted. The results showed a significant decrease of primordial follicles in *Zp3‐Cre;iDTR* ovaries compared to controls with DT treatment (Figure 3E). After 10 weeks of treatment, only (256 ± 28) primordial follicles were left in the ovaries of *Zp3‐Cre;iDTR* ovaries, which was in sharp contrast to the number of primordial follicles (880 ± 133) in normal ovarian reserve of control ovaries. Furthermore, the levels of follicle‐stimulating hormone (FSH) in serum, which was one of the major detecting index in POI patients, were significantly increased at 11 weeks in the serum of DT‐treated adult *Zp3‐Cre;iDTR* females compared to that of the control (2.50 ± 0.36 vs 0.47 ± 0.22 ng/mL) (Figure 3F). These results showed that the *Zp3‐Cre;iDTR* mouse model successfully mimicked the symptoms of POI patients after 10 weeks of DT treatment, and the resulting mice were referred to as iPOI (inducible POI) mice in our study.

### 2.5 EGF functions to improve primordial follicle activation in iPOI mice

**FIGURE 4:**
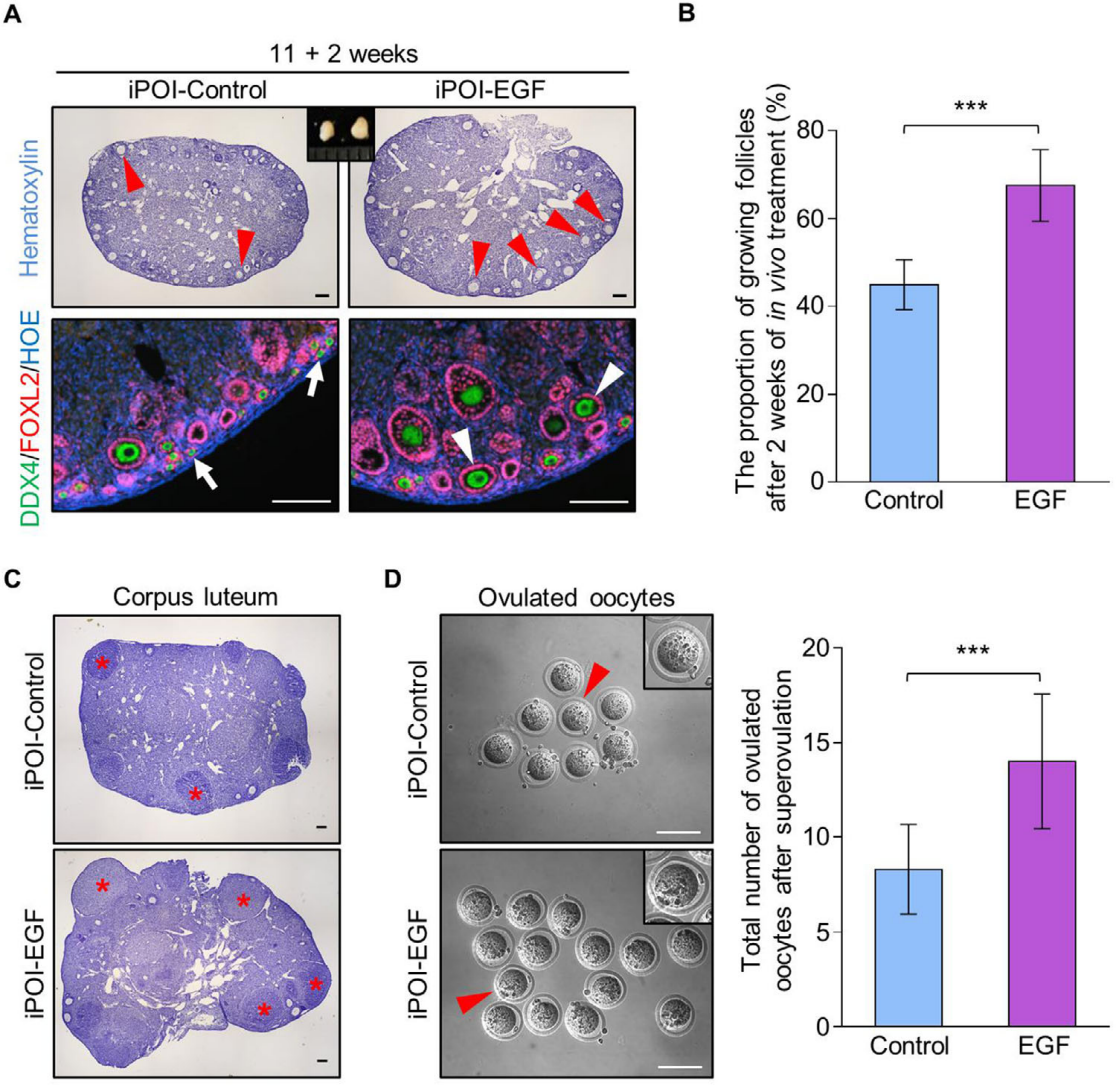
In vivo EGF administration improves primordial follicle activation and the oocyte retrieval rate from iPOI ovaries. (A) The bilateral ovaries from each iPOI female were covered by Matrigel with or without EGF through ovarian bursa injection. After 2 weeks of treatment, a markedly increased number of growing follicles (arrowheads) was observed in the cortical regions of EGF‐treated ovaries compared to that of the control ovaries with majority of primordial follicles (arrows) (green: DDX4, purple: FOXL2, blue: Hoechst, *n* = 3). (B) Follicle counting results showed a significant increase in growing follicles in EGF‐treated ovaries compared to that of the controls (68 ± 7% vs 45 ± 5%) (*n* = 6). (C) Three weeks after EGF treatment, superovulation was performed to detect the oocyte retrieval rate in iPOI females. Histological analysis showed a markedly increased number of corpus luteum (asterisks) in EGF‐treated ovaries compared to controls (*n* = 13). (D) A significantly increased number of ovulated oocytes after superovulation was collected from EGF‐treated ovaries compared to that of controls (14 ± 4 vs 8 ± 2) (*n* = 13). In B and D, data represent the mean ± SD of biological triplicate experiments. ***P < .001, by two‐tailed unpaired Student's *t* test. Scale bars: 100 µm

Utilizing the established iPOI mouse model, we next investigated whether in vivo treatment of EGF could stimulate the follicle activation in iPOI mouse ovaries. To minimize the variation of ablating efficiency among female mice, the bilateral ovaries from each iPOI female were injected with gel containing EGF or gel only separately. In contrast to the control ovary, in which the majority of primordial follicles (Figure 4A, arrows) existed in the ovary after 2 weeks of treatment, histological analysis showed a markedly increased distribution of growing follicles in the ovary of the EGF‐treated side (Figure 4A, arrowheads). The follicle‐counting results confirmed that there was a significantly increased ratio of growing follicles in EGF‐treated ovaries compared to that of controls (68 ± 7% vs 45 ± 5%) (Figure 4B), showing that in vivo EGF treatment efficiently improved the activation of dormant primordial follicles in iPOI ovaries.

We next detected whether EGF‐activated follicles were functional to ovulate and produce the mature eggs. Superovulation was performed on iPOI animals after 3 weeks of treatment with or without EGF. Histological analysis showed a markedly increased number of corpus luteum (Figure 4C, asterisks) in EGF‐treated ovaries compared to those of controls. Meanwhile, a significantly increased number of ovulated oocytes (Figure 4D) were harvested from oviducts of EGF‐treated side in each female compared to controls (14 ± 4 vs 8 ± 2). This result indicated that in vivo EGF treatment significantly improved the oocyte retrieval rate of iPOI ovaries, showing a prospective application of EGF in the treatment of patients with POI in clinical practice.

### 2.6 EGF treatment improves the activation and development of human follicles

**FIGURE 5:**
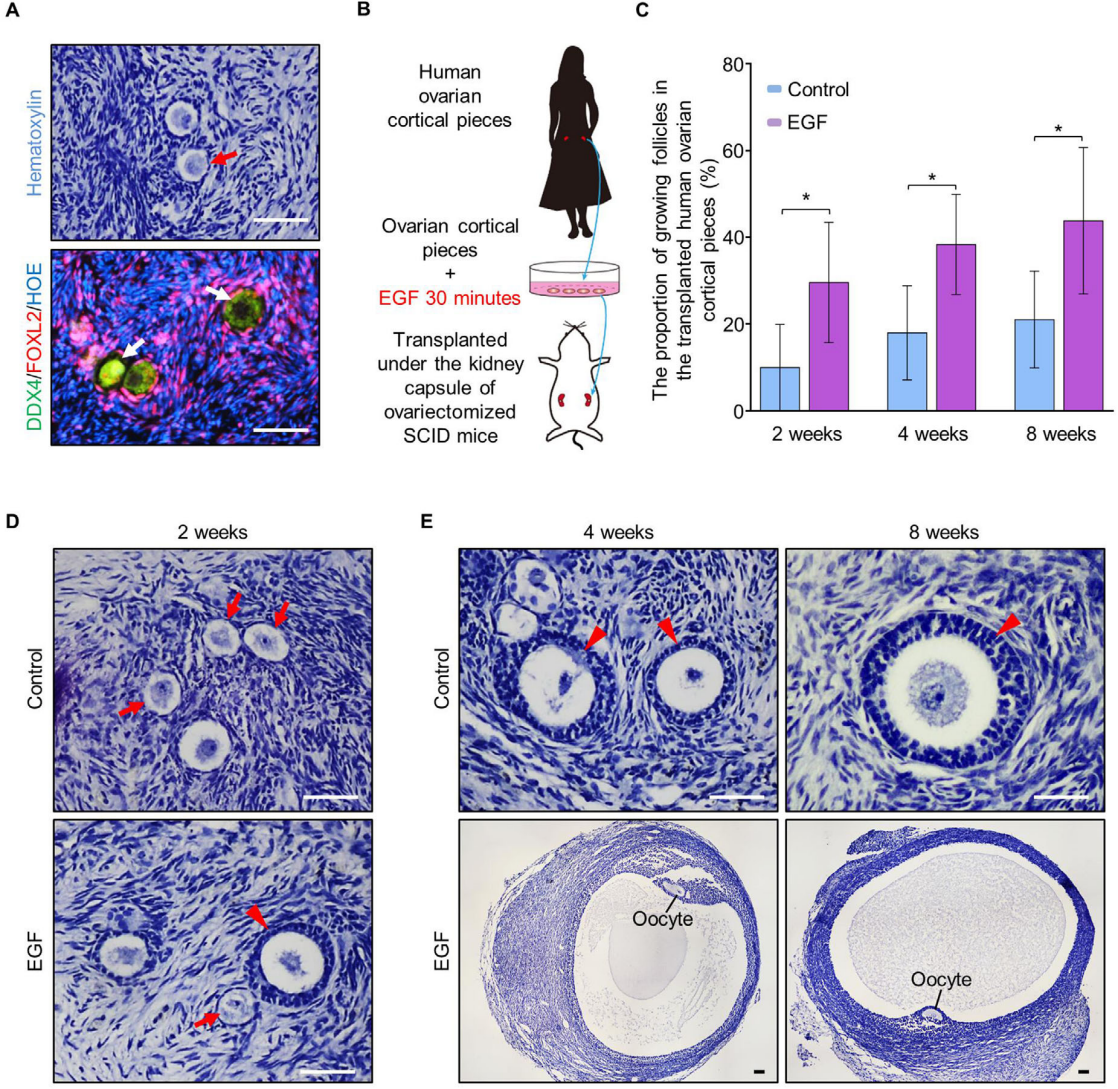
EGF treatment improves the activation and development of human follicles. (A) Histological and immunostaining detection to test the existence of primordial follicles in fresh human ovarian pieces before transplantation (green: DDX4, purple: FOXL2, blue: Hoechst, *n* = 5). (B) Detecting the effect of EGF on stimulating human dormant follicle activation. Human ovarian cortical pieces which contained a large number of primordial follicles were cut into small cubes (1 mm^3^) and incubated with or without EGF for 30 minutes in dishes, then the treated tissues were xenografted into the kidney capsule of ovariectomized SCID mice for further development. (**C**) After 2 weeks (30 ± 12% vs 10 ± 9%), 4 weeks (38 ± 10% vs 18 ± 10%) and 8 weeks (44 ± 15% vs 21 ± 10%) of in vivo development under kidney capsule, follicle counting showed a significantly increased proportion of growing follicles in EGF‐treated human ovarian pieces compared to that of the controls (*n* = 5 per group). (D) After 2 weeks of transplantation, secondary follicles with multilayer granulosa cells (arrowhead) were observed in EGF‐treated ovarian pieces, and only primary follicles were found in the control group (*n* = 5). (E) Preovulatory follicles were found in EGF‐treated ovarian pieces after 4 and 8 weeks of transplantation, whereas only the secondary follicles were found in the control group (*n* = 5). Data represent the mean ± SD of biological triplicate experiments. *P < .05, by two‐tailed unpaired Student's *t* test. Scale bars: 50 µm

Finally, we extended our study to test whether EGF also functioned to improve the activation of dormant primordial follicles in women. Human ovarian pieces were collected during laparoscopic ovarian wedge resection from six patients with polycystic‐ovary‐syndrome (PCOS) with aged ranging from 25 to 35. The cortex of ovaries was cut into small cubes (approximately 1 mm^3^), and the profiles of follicle distribution in fresh samples of all patients were detected to ensure the abundance of primordial follicles in every samples (Figure 5A and Supplemental Table S1). As a result, five qualified patients in totally six were introduced into the study, and the samples from each patient were cut into around 35 pieces which were separated to incubate with or without EGF for 30 minutes. Therefore, totally 75 treated pieces and 75 control pieces (15 treated pieces and 15 control pieces in each patient, three pieces in each animal) were xenografted into the kidney capsules of ovariectomized SCID (severe combined immunodeficiency) mice for further development (Figure 5B).

To investigate whether EGF improved the activation and development of human follicles, we quantified the follicle developmental profile in ovarian pieces after 2, 4, and 8 weeks of transplantation. Although the activating ratio was not stable in different patients, the proportion of growing follicles were significantly increased in EGF‐treated grafts at all detected time points in all patients compared to the controls (Table 1). Statistical analysis showed a significant increase in the growing follicle proportion after EGF treatment compared to that of the control (Figure 5C, 2 weeks, 30 ± 12% vs 10 ± 9%; 4 weeks, 38 ± 10% vs 18 ± 10%; 8 weeks, 44 ± 15% vs 21 ± 10%).

**TABLE 1:** The proportion of growing follicles in the transplanted human ovarian cortical pieces

		Patient 1	Patient 2	Patient 3	Patient 4	Patient 5	Mean ± SD
2 weeks	Control	15%	24%	6%	‐1%	5%	10 ± 9%
	EGF	29%	37%	16%	17%	49%	30 ± 12%
4 weeks	Control	22%	9%	4%	24%	30%	18 ± 10%
	EGF	47%	44%	23%	30%	48%	38 ± 10%
8 weeks	Control	18%	19%	15%	13%	41%	21 ± 10%
	EGF	48%	38%	23%	41%	69%	44 ± 15%

Human cortical ovarian pieces were collected from five patients who had PCOS. The tissues were cut into small cubes and incubated with or without EGF for 30 minutes. After being xenografted into the kidney capsule of ovariectomized SCID mice, the follicular distributions in different groups were quantified at 2, 4, and 8 weeks of in vivo development. To statistic the change of follicle activating proportions, each data from individual patient was normalized by the original activating ratio in this patient from fresh tissue before transplantation. Data represent the mean ± SD of biological triplicate experiments.

Moreover, histological analysis showed that many secondary follicles (Figure 5D, arrowheads) with multilayer of granulosa cells were observed in EGF‐treated ovarian pieces after 2 weeks of transplantation, whereas most of the follicles remained in primordial or primary stages (Figure 5D, arrows) in controls. After 4 and 8 weeks of transplantation, although some follicles at secondary stages were observed, no antral follicles were found in any of the control grafts (Figure 5E). This was in sharp contrast to that preovulatory follicles were observed in EGF‐treated ovarian pieces after both 4 and 8 weeks of treatment (Figure 5E), showing that EGF treatment also improved the development of human follicles. Therefore, we concluded that in vitro EGF treatment is an efficient approach to improve the development of human follicles after xenotransplantation, indicating that EGF might be a potent functional stimulator for human infertility treatment.

## 3 DISCUSSION

As the most abundant fertility resource in women, dormant primordial follicles have attracted extensive attention as a novel target to overcome female infertility in the past decade.^30^ However, the inadequate understanding of early follicle development, especially the unknown of upstream mechanisms and extra‐follicle activating factors in regulating the activation of dormant primordial follicles under physiological conditions, makes the utilization of those natural reproductive resources in clinical application difficult. Meanwhile, the inconvenience of the current strategy of the IVA of dormant primordial follicles also increased the difficulty of widespread application of such technology.^10^ In the current study, by testing the activating efficiency of several endogenous ovarian growth factors, we found that EGF was a potent upstream functional factor that stimulated the activation of primordial follicles in both human ovarian cortical pieces and mouse ovaries. Moreover, based on the characteristics of primordial follicle distribution on the cortical region of adult ovaries, an in vivo activating strategy was established in our study by combining EGF and a bio‐gel carrier to cover the ovarian surface. Our results supplied direct experimental data showing that ovarian topical administration of the follicle‐activating drug EGF improved the activation and development of follicles in the murine ovaries of normal and induced premature ovarian failure model mice, which represented a possibility of a novel infertility treatment strategy for both aged women and women with ovarian diseases.

Although various growth factors have been reported to function in regulating follicle development, our results showed that EGF was the most efficient one to improve the activation of dormant primordial follicles in our system. Previous reports showed that EGF was a rapid activator of CDC42 in various cell lines and triggered the downstream signaling activity including the PI3K pathway.^7^, ^24^, ^31^ This was consistent with our result that EGF treatment improved the activation of CDC42‐PI3K signaling in ovaries. Meanwhile, EGF not only enhanced the activation of primordial follicles but also improved the development of growing follicles. As a fundamental growth factor that regulates various cellular events, EGF is involved in processes related to folliculogenesis and ovarian development, such as increasing the proliferation of both granulosa cells and ovarian stromal cells,^16^, ^18^, ^32^ and improving the maturation of oocytes.^33^ In the granulosa cells, it was well known that EGF and EGF‐like growth factors play as a key upstream regulator to stimulate several key pathways including ERK1/2 pathway and PI3K pathway to trigger the oocytic meiotic resumption.^34^, ^35^, ^36^ Meanwhile, EGF was also reported to improve the MAPK and PI3K pathways and enhance the proliferation of the ovarian stromal cells.^18^ Considering the strategy of drug delivery in our system, there is no doubt that EGF improves the development of both follicle cells and nonfollicle stromal cells in the ovary after treatment. These data suggested that the mechanisms of EGF's beneficial effects were complex in the ovary. This is in consistent to the phenotype of our in vivo activation in female fertility. Not like the previously reported phenotypes of primary ovarian insufficiency in various genetically modified mouse models, such as oocyte deletion of *Pten* ^37^ or GC deletion of *Rptor*,^12^ our in vivo activation only led to an acute increase of growing follicle proportion in the ovaries, but there was no effect on the long‐term fertility in treated females. These results indicated that the in vivo EGF treatment only caused a mild activating effect which would not lead to an overall activation of dormant primordial follicles, and the balance between growing follicles and dormant follicles is reestablished after the acute change of ovarian microenvironment by EGF treatment.

As the cornerstone of classic assisted reproductive technology, the convenience of in vivo gonadotrophin treatment to stimulate the development and ovulation of existing antral follicles allowed the current IVF technique to be a routine practice in infertility treatment.^38^ Unlike the antral follicles that respond to hormones, dormant primordial follicles are believed to be independent on endocrine regulation.^5^ Therefore, it is hard to selectively awaken the dormant primordial follicles through a traditional drug delivery approach. In our study, we tested the effect of ovarian topical administration of EGF through a carrier bio‐gel to stimulate the activation of primordial follicles in vivo in mice. With this strategy, theoretically all remaining dormant follicles that localized on ovarian surface could be stimulated by drugs, and the efficiency of activation should be increased greatly with a minimized side effects. Indeed, our results revealed that the activation of primordial follicles was significantly improved in both normal and iPOI ovaries. Based on our results, we believed that the ovarian surface drug delivery approach should be a flexible strategy to affect the female reproduction. Therefore, the current study supplied a conceptual strategy which might improve the female reproductive healthy in the future.

Certainly, there is still a long way till the application of such in vivo activation approach to be performed in clinical practice. Although EGF is a well‐studied endogenous factor in the ovary, the physiological extra‐follicle mechanisms that governing the activating primordial follicles are still remaining elusive. What factors and which cell types involved in the surrounding microenvironment to control the fate of primordial follicles are also unknown. For in vivo activation, or in vivo regulating the development of dormant primordial follicles, there is still a great room for improvement in both the stimulating drugs and the carriers of drug delivery. For example, a modification of EGF to make it sustained action for a long release period will definitely increase the efficiency of in vivo activation. Although it is still unknown whether the current approach is effective in humans, we believe that in vivo activation of primordial follicles by topical administration will be a potential novel ART strategy with great development prospects.

## 4 METHODS

### 4.1 Animals

The C57BL/6J mice and SCID mice were obtained from the Laboratory Animal Center of the Institute of Genetics (Beijing). *Zp3‐Cre* mice were provided by Prof. Meijia Zhang from China Agricultural University, Beijing, China. *iDTR* mice (007900) were purchased from Jackson Laboratory. The *Zp3‐Cre;iDTR* growing follicle‐ablation model was generated by crossing *Zp3‐Cre* transgenic males with *iDTR* knock‐in females. In *iDTR* female mice, *loxP*‐flanked upstream *STOP* sequence blocks the DTR expression in cells. When bred to *Zp3‐Cre* transgenic male mice, the STOP sequence is deleted in the Cre positive oocytes of all growing follicles to active the DTR expression. Therefore, all growing oocytes expressing DTR are able to be ablated following diphtheria toxin (DT, 1 µg/mL, Sigma, USA) administration as illustrated in Figure 3A. To selectively eliminate the existing growing follicles, *Zp3‐Cre;iDTR* female mice were intraperitoneally injected with DT (10 µg·kg^−1^ BW) from 1 week to 11 weeks after birth. The half‐time of DT is less than 8 hours in vivo.^39^ Mice were housed in mouse facilities that conformed to the standards and requirements of the Institutional Animal Care and Use Committee of China Agricultural University.

### 4.2 Ovary culture in vitro and kidney capsule transplantation

Paired ovaries were collected from postnatal day 6 (PD 6) female mice. Unilateral ovary from one female was incubated with EGF (100 ng/mL, E4127, Sigma, USA) for 30 minutes in 24‐well culture dishes (NEST, China) with an insert (PITP01250, Millipore, USA), and ovary in the other side served as a control. The DMEM/F12 nutrient mixture (GIBCO, Life Technologies, USA) was supplemented with penicillin/streptomycin as the culture media. After the treatment, the ovaries were transplanted under the kidney capsules of bilaterally ovariectomized adult females. After 2 weeks of transplantation, mice were sacrificed to detect follicular development. For immunofluorescence staining or Western blot, ovaries were cultured with different factors GDF‐9 (500 ng/mL, 739‐G9/CF, R&D Systems, USA), SCF (500 ng/mL, 455‐MC/CF, R&D Systems, USA), or EGF for 30 minutes, respectively, and followed a 12‐hours culture in drug‐free medium. Then the samples were harvested for experiments.

### 4.3 Human ovarian cortex culture and kidney capsule transplantation

Human ovarian samples were obtained from five patients with PCOS receiving laparoscopic ovarian wedge resection treatment in the Center for Reproductive Medicine of Shandong University. Ovarian fragments from each patient were carefully cut into small cubes (1 mm^3^). A part of cortical pieces was fixed for histological examination, and the remaining cubes were cultured in DMEM/F12 plus penicillin‐streptomycin solution with or without EGF on the insert in 24‐well culture dishes for 30 minutes. The media was added so that it covered the cubes with a thin layer. After culturing for 30 minutes, the cortical cubes were xenografted under the kidney capsule of ovariectomized adult SCID mice. Every mouse was transplanted with only ovarian pieces from one patient, and total 25 mice were used in this experiment. Mice were sacrificed at 2, 4, and 8 weeks after transplantation, and the human samples were collected to detect. All experiments were approved by the Institutional Review Board of Reproductive Medicine of Shandong University and informed consent was obtained from all participant patients.

### 4.4 Immunofluorescence staining

Samples were fixed in 4% paraformaldehyde solution in PBS (PFA, sc‐281692, Santa Cruz Biotechnology, USA) overnight, embedded in paraffin and sectioned to obtain 8‐µm serial paraffin sections. The ovarian sections were processed to deparaffinize and rehydrate. The antigen of ovarian sections was retrieved by high temperature (95‐98°C) for 16 minutes in sodium citrate buffer (pH 6.0). Then, the sections were blocked for 60 minutes at room temperature and incubated with different primary antibodies at 4°C for 12 hours. The primary antibodies were as follows: FOXO3a antibody (12829, rabbit, 1:300, Cell Signaling Technologies, USA), FOXL2 antibody (IMG‐3228, goat, 1:300, Novus, USA), and DDX4 antibody (ab27591, mouse, 1:400, Abcam, UK). Subsequently, the ovarian sections were washed with PBS and incubated with Alexa Fluor 555‐ or 488‐conjugated donkey secondary antibody (1:100, Life Technologies, USA) at 37°C for 60 minutes and were counterstained with Hoechst 33342 (1:100, Beyotime, China). The slides were checked and photographed by using the Nikon Eclipse Ti digital fluorescence microscope.

### 4.5 Western blot

Cultured mouse ovaries were lysed in WIP lysis solution (Cell Signaling Technologies, USA). Sample proteins were separated by electrophoresis by 10% SDS‐PAGE and transferred to PVDF (polyvinylidene fluoride) membranes (IPVH00010, Millipore, USA). Then, the membranes were blocked with 5% nonfat‐dry milk for 60 minutes and incubated at 4°C overnight with the following primary antibodies. The primary antibodies were as follows: p‐FOXO3a (9466, 97 kDa, 1:1000, Cell Signaling Technologies, USA), FOXO3a (12829, 82‐97 kDa, 1:1000, Cell Signaling Technologies, USA), p‐AKT (4060, 56 kDa, 1:1000, Cell Signaling Technologies, USA) and AKT (4691, 56 kDa, 1:1000, Cell Signaling Technologies, USA). The membranes were washed thoroughly with tris‐buffered saline with tween and incubated with the appropriate secondary antibody (1:5000, ZSGB‐BIO, China). The level of β‐actin (42 kDa, 1:1000, Sigma, USA) was used as an internal control. The membranes were visualized by the SuperSignal detection system (Prod 34080, Thermo, USA). To quantify the results of immunoblot, the image was quantified using Image J software.

### 4.6 CDC42 activity assay

CDC42 activity was determined using the CDC42 Activation Assay Biochem Kit™ (BK034‐S, Cytoskeleton, USA). Clarified ovarian lysates were incubated in a microfuge tube with PAK‐PBD beads for 1 hour at 4°C on rotator. The supernatant was very carefully removed, after the PAK‐PBD beads were pelleted by centrifugation at 4000*g* at 4°C for 1 minute. The beads were washed once with 500 µL of wash buffer. The PAK‐PBD beads were pelleted by centrifugation at 4°C at 4000*g* for 3 minutes. The supernatant was very carefully removed. Next, 20 µL of 2 × Laemmli sample buffer was added to each tube to thoroughly resuspend the beads. The bead samples were boiled for 2 minutes. The samples were analyzed by Western blot using an anti‐CDC42 antibody (1:250). The amount of GTP‐CDC42 (PAK‐PBD‐bound CDC42) indicated the CDC42 activity. The total CDC42 present in each sample was determined by Western blot.

### 4.7 Histological analysis

Ovaries were fixed in 4% cold PFA for 12 hours and processed to obtain serial paraffin sections at 8 µm thickness. Every fifth ovarian section stained with hematoxylin (sc‐24973A, Santa Cruz Biotechnology, USA) was counted for the number of primordial and primary follicles. Then, the counting numbers were multiplied by five to calculate the number of all primordial or primary follicles. Every section was counted for the presence of secondary and antral follicles. Only the follicles with oocytes were counted to exclude the effect of residual structures after oocyte ablation. For human follicle counting, every section was analyzed to calculate the number of all follicles. The proportion of growing follicles in fresh tissues in each patient was subtracted to normalize the final proportion of growing follicles at 2, 4, and 8 weeks after transplantation.

### 4.8 Ovarian topical administration in vivo

To perform the ovarian typical administration, the females were anesthetized with avertin (300 mg/kg) (T48402, Sigma, USA) and the ovaries were exposed from the incisions on the backs gently, as illustrated in Supplemental Figure S2A. EGF (100 ng/mL) was dissolved in the liquid growth factor reduced Matrigel (354230, BD, USA) on ice or hyaluronan (5 mg/mL, CE0197, Bioregen, China) and injected into the ovarian bursa using a microinjection arm, as illustrated in Figure 2A. For regular females, gel with or without EGF were given into bilateral ovarian bursas. For iPOI animals, unilateral ovarian bursa was given gel with EGF, and the other was injected with gel only to be the control. After matrigel solidification at body temperature, the incisions were sutured.

### 4.9 Oocyte collection and counting

After 3 weeks of EGF treatment, *Zp3‐Cre;iDTR* females were intraperitoneally injected PMSG (500 IU/kg) for superovulation. After 46‐48 hours, human chorionic gonadotrophin (500 IU/kg) was intraperitoneally injected. Oocytes were collected from oviducts at 16 hours after injection. The cumulus cells were separated by pipetting in EmbryoMax M2 medium with hyaluronidase (MR‐051‐F, Millipore, USA). The number of ovulated oocytes was counted.

### 4.10 Fertility testing

Mating trials were initiated at 1 week after surgery. Healthy males at 8‐10 weeks of age were mated with treated females (1:2) for 6 months. The number of pups from each female was recorded twice per week to analyze the reproductive curve.

### 4.11 Serum hormone measurement

Orbital blood was obtained under anesthetized conditions with avertin. Serum was separated by centrifugation at 3000 rpm at 4°C and stored at −20°C. The levels of FSH were measured by the immunoassay kits (FSH (Rodent) ELISA Kit, KA2330, Abnova). The optical density at 450 nm was read with a microtiter well reader. A standard curve was constructed to calculate the corresponding concentration of FSH for each specimen by plotting a graph of the absorbance of each reference standard.

### 4.12 Statistical analysis

The experiments were repeated at least three times. Experimental data were expressed as the means ± SD with each experiment, analyzed by *t*‐test and considered statistically significant at values of *P* < .05.

## STUDY APPROVAL

All animal studies were conformed to the approval of the Institutional Animal Care and Use Committee of China Agricultural University. The collection and use of human ovarian samples were conducted with ethics approval of the Ethics Committee Review Board of Reproductive Medicine, Shandong University.

## COMPETING INTERESTS

Jiawei Zhang, Lei Yan, Yibo Wang, Shuo Zhang, Xueqiang Xu, Yanli Dai, Shidou Zhao, Zhen Li, Yan Zhang, Guoliang Xia, Yingying Qin, and Hua Zhang declare that they have no conflict of interest.

## AUTHOR CONTRIBUTIONS

JZ, YQ, and HZ designed research; JZ, LY, YW, SZ, XX, DY, and YZ performed research; JZ, LY, YW, ZL, YZ, and HZ analyzed data; JZ and HZ wrote the paper. All authors have seen and approved the final version.

## Supporting information

FigureS1.jpg

FigureS2.jpg

FigureS3.jpg

FigureS4.jpg

SuppMat.docx

## Data Availability

All data generated or analyzed during this study are included in this published article and its supplementary information files.
