# Supplementary figures and images for: In vivo and in vitro activation of dormant primordial follicles by EGF treatment in mouse and human
Source: Clin Transl Med. 2020 Sep 27;10(5):e182. doi: 10.1002/ctm2.182 (PMC7520080; doi:10.1002/ctm2.182)

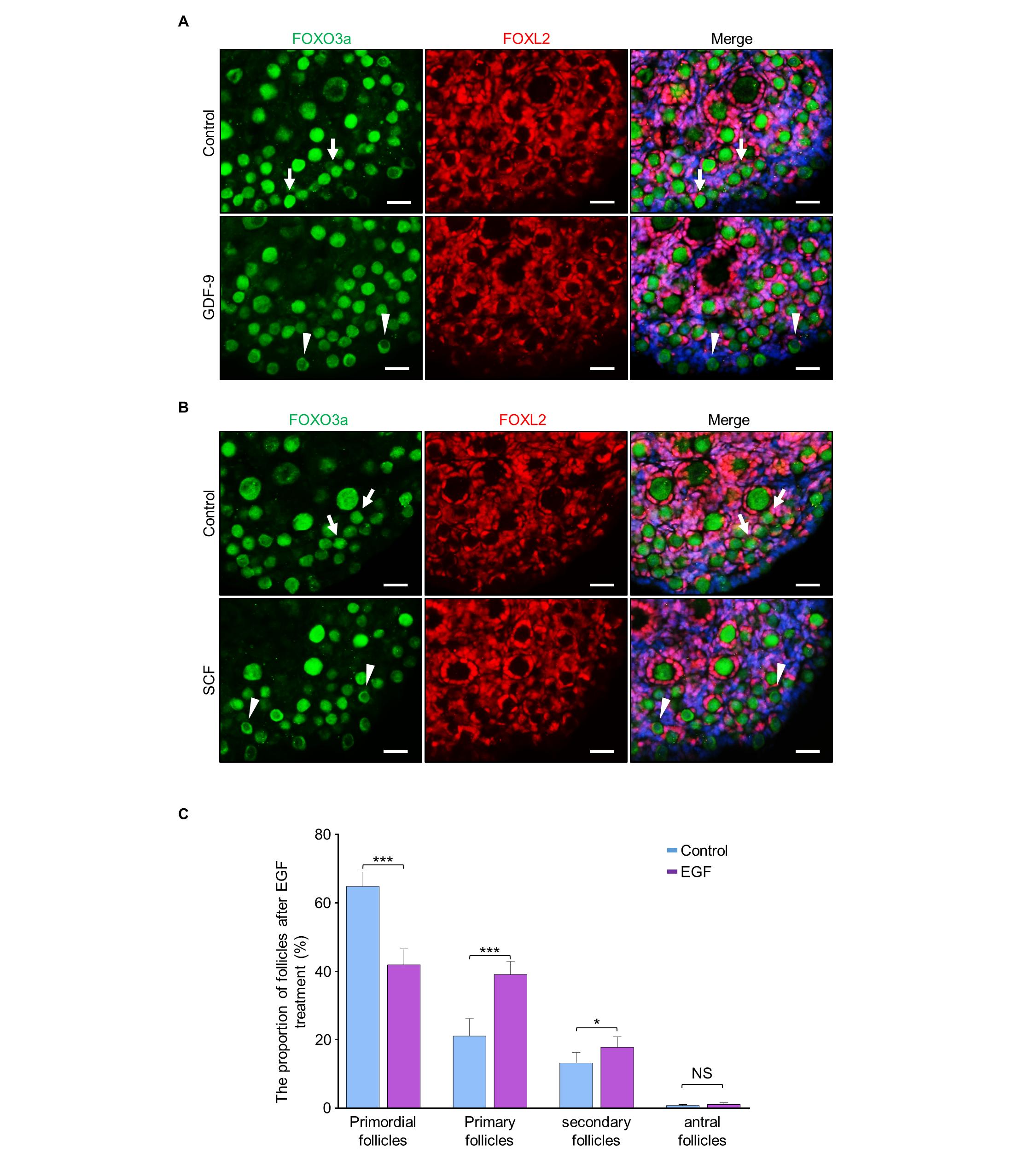

Supplement: Supplementary file 1 — FigureS1.jpg [file CTM2-10-e182-s001.jpg]

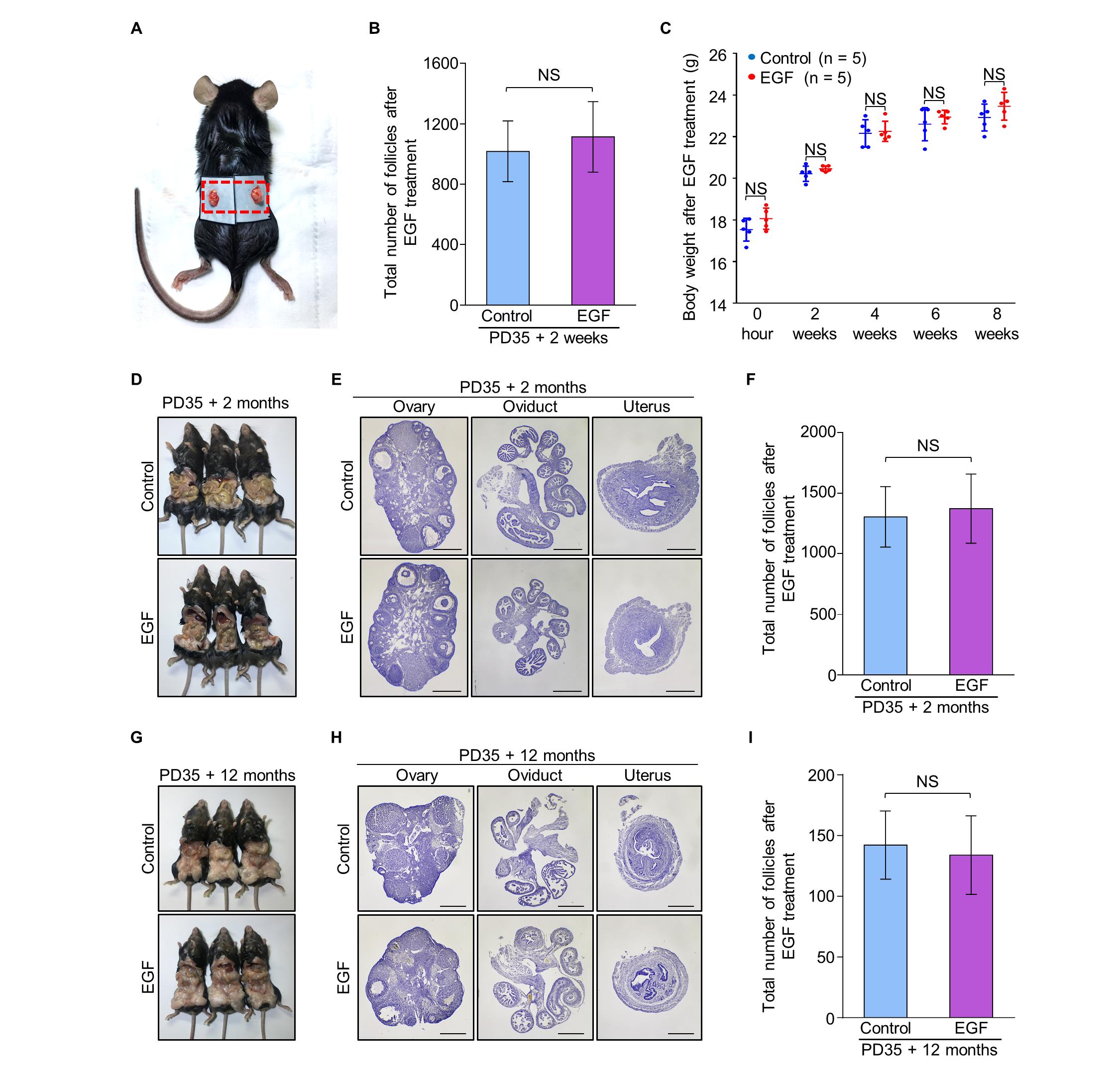

Supplement: Supplementary file 2 — FigureS2.jpg [file CTM2-10-e182-s002.jpg]

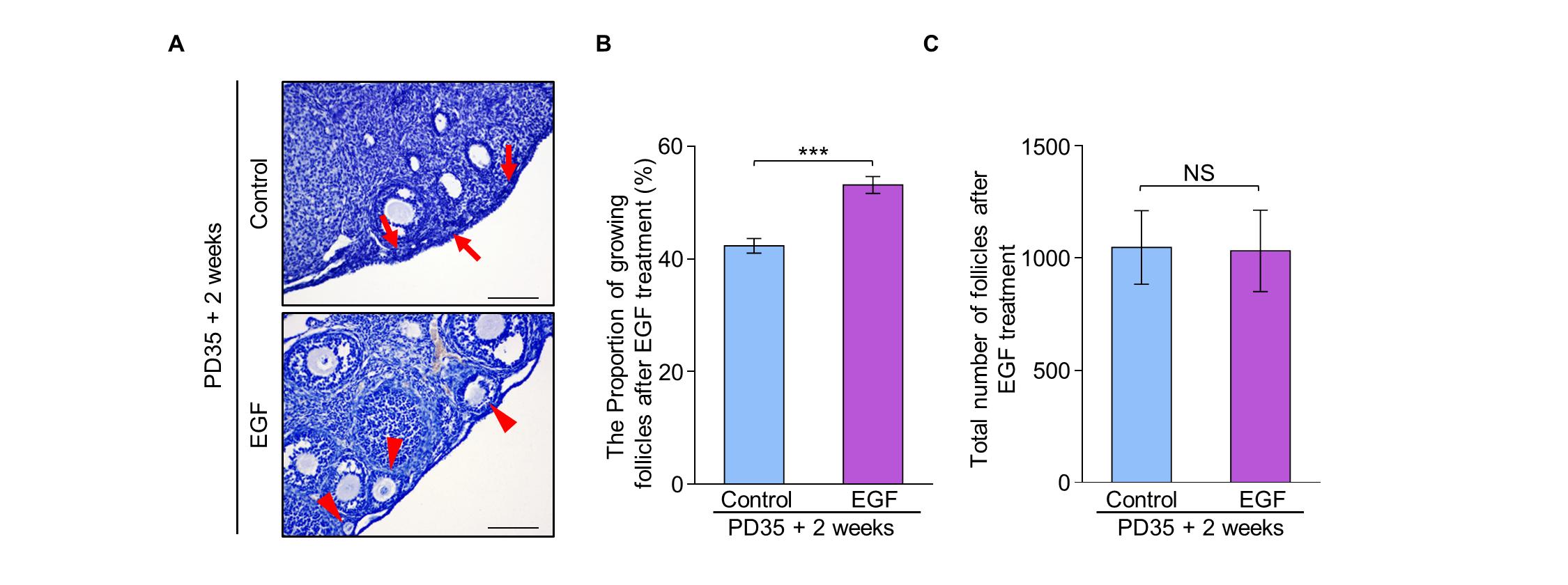

Supplement: Supplementary file 3 — FigureS3.jpg [file CTM2-10-e182-s003.jpg]

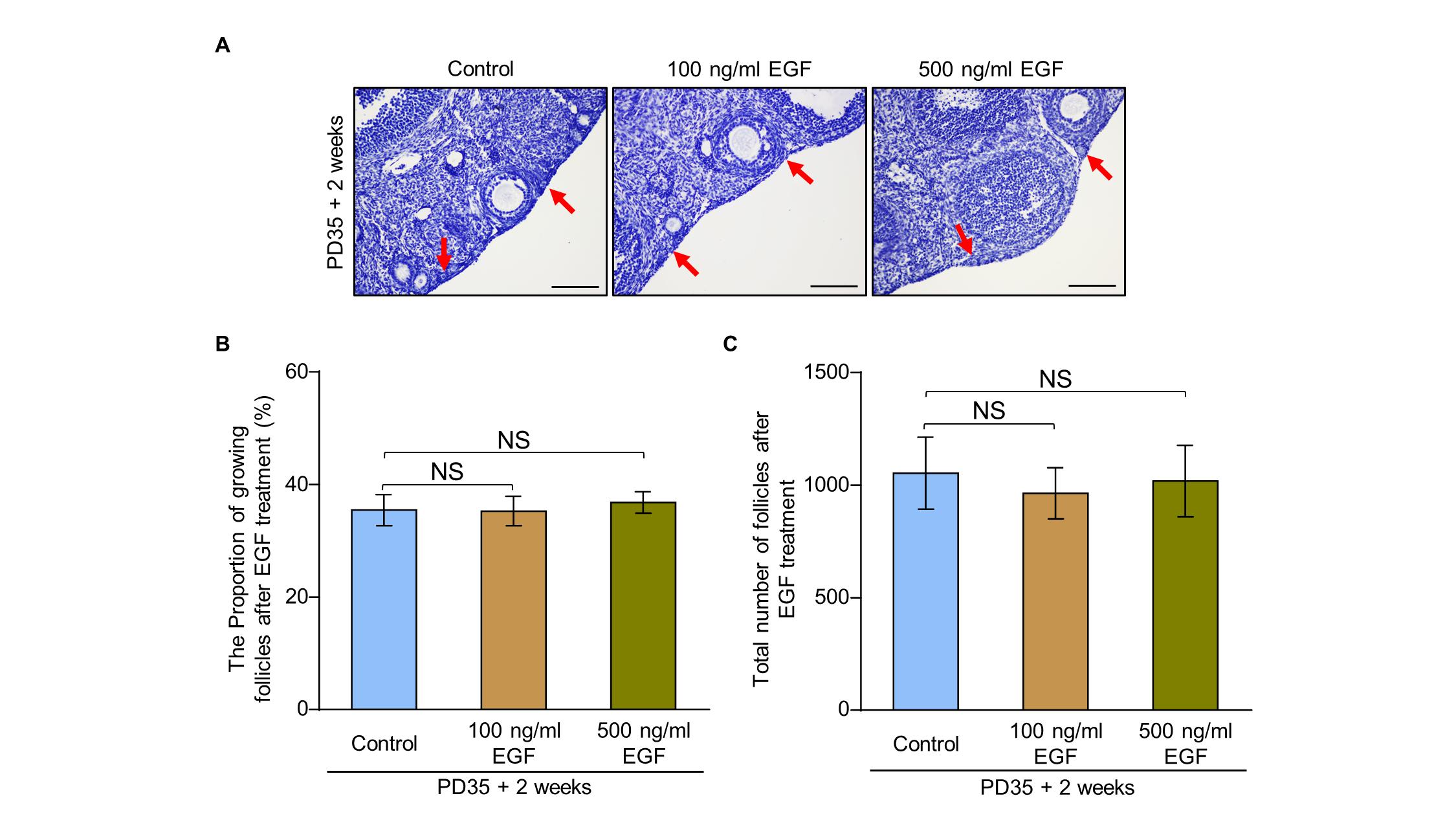

Supplement: Supplementary file 4 — FigureS4.jpg [file CTM2-10-e182-s004.jpg]
