# Supplementary material for: In vivo and in vitro activation of dormant primordial follicles by EGF treatment in mouse and human
Source: Clin Transl Med. 2020 Sep 27;10(5):e182. doi: 10.1002/ctm2.182 (PMC7520080; doi:10.1002/ctm2.182)
Supplement: Supplementary file 5 — SuppMat.docx [file CTM2-10-e182-s005.docx]

**Supplemental material**

***In vivo* and *in vitro* activation of dormant primordial follicles by EGF treatment in mouse and human**

**Jiawei Zhang^1^, Lei Yan^2^, Yibo Wang^1^, Shuo Zhang^1^, Xueqiang Xu^1^, Yanli Dai^1^, Shidou Zhao^2^, Zhen Li^3^, Yan Zhang^1^, Guoliang Xia^1^, Yingying Qin^2^*****, Hua Zhang^1^***

1. State Key Laboratory of Agrobiotechnology, College of Biological Sciences, China Agricultural University, Beijing 100193, China.
2. Center for Reproductive Medicine, Shandong University, Jinan 250021, China.
3. State Key Laboratory of Plant Physiology and Biochemistry, College of Biological Sciences, China Agricultural University, Beijing 100193, China.

*Corresponding author: YYQ, [qinyingying1006@163.com](mailto:qinyingying1006@163.com); HZ, huazhang@cau.edu.cn

**Running title: *In vivo* activation of dormant follicles by EGF**

**Key words:** primordial follicle activation; EGF; premature ovarian insufficiency; *in vivo* activation; non-invasive administration

This file includes:

Supplementary Figure S1

Supplementary Figure S2

Supplementary Figure S3

Supplementary Figure S4

Supplementary Table S1

**Supplementary Materials**

**Figure S1**

**
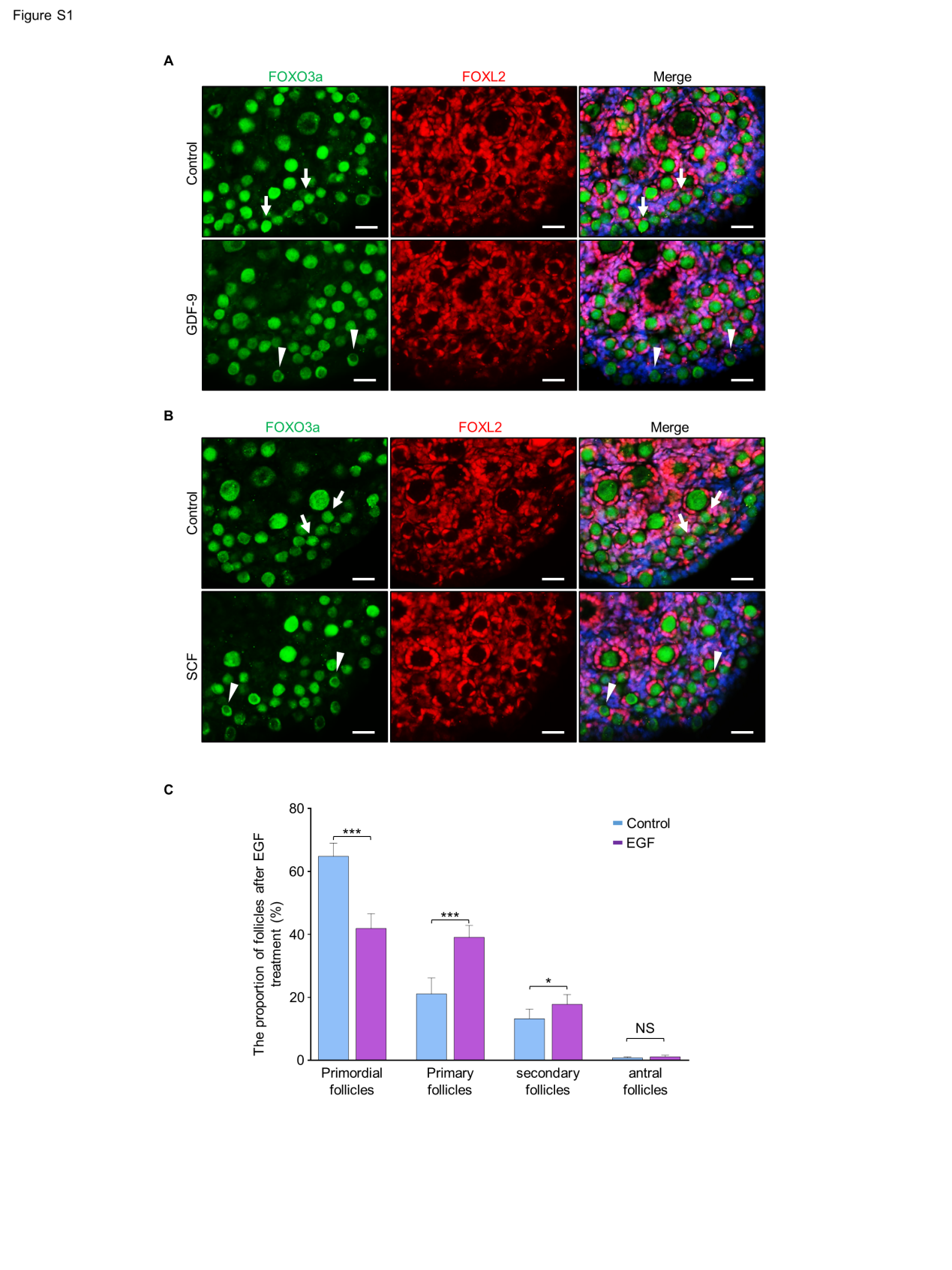
**

**Figure S1. The effect of GDF-9, SCF and EGF treatment on stimulating the activation of primordial follicles in mouse ovaries.** (**A-B**) Intact ovaries from PD 6 females were isolated and cultured with or without factors (GDF-9 or SCF) for 30 minutes, followed by culture in factor-free medium for 12 hours. After culture, the expression of FOXO3a was detected by immunofluorescence staining (green: FOXO3a, purple: FOXL2, blue: Hoechst). FOXO3a localized to the nuclei (arrows) of dormant oocytes and shuttled to the cytoplasm (arrowheads) of activated oocytes. (**C**) Distribution of follicles at different stages in the ovaries of PD6 mice after EGF treatment (n = 5), showing a significant increased propotion of primary follicle in the EGF treated ovaries. The experiments were repeated at least three times, and representative images are shown. NS, *P* > 0.05, **P* < 0.05 and ****P* < 0.001, by 2-tailed unpaired Student’s t test. Scale bars: 25 μm.

**Figure S2**


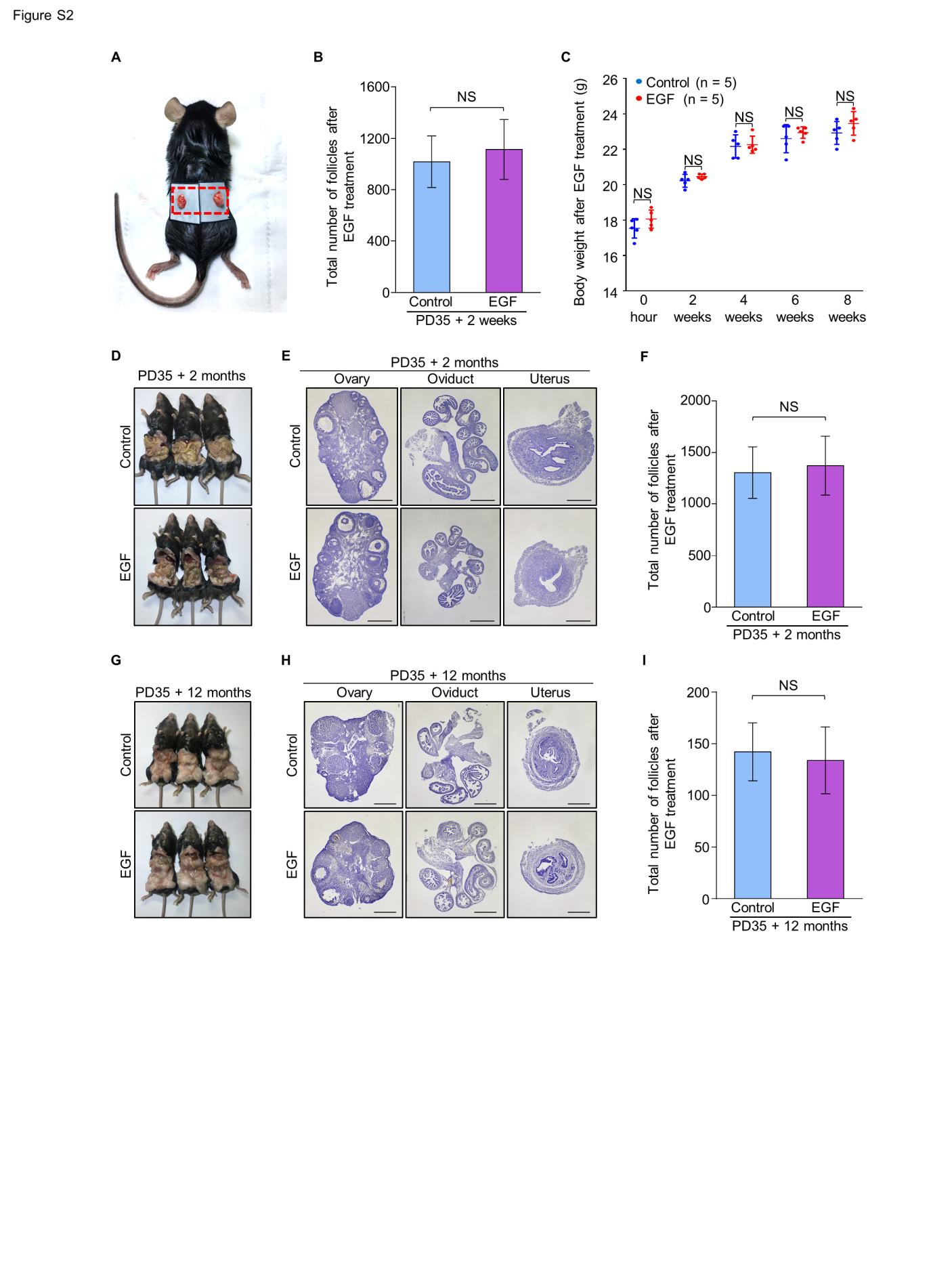
**Figure S2. EGF treatment has no effect on the health and fertility of treated females.** (**A**) The mice after surgery of ovarian topical EGF-Matrigel administration showed the ovaries out of the body. (**B**) After two weeks of EGF treatment, follicle counting showed an identical number of total follicles in EGF-treated ovaries compared to that of the controls (1114.17 ± 212.92 v.s. 1018.33 ± 183.41) (n = 3). (**C**) A comparable increase of body weight in EGF-treated and control animals during 8 weeks of detection (n = 5 per group). (**D-I**) The long-term side effects of EGF treatment on the health and fertility of EGF-treated females were investigated. Anatomy analysis showed no signs of carcinogenesis in animals after 2 months (**D**) and 12 months (**G**) of EGF treatment. Histological analysis revealed normal development of the ovary, oviduct and uterus at 2 months (**E**) and 12 months (**H**) in EGF-treated mice. An identical number of total follicles in EGF-treated and control ovaries at both 2 months (1372.17 ± 259.54 v.s. 1304.17 ± 227.81) (**F**) and 12 months (133.83 ± 31.01 v.s. 145.20 ± 35.95) (**I**) after treatment (n = 3). In **B**, **C, F** and **I**, data represent the mean ± SD of biological triplicate experiments. NS, *P* > 0.05, by 2-tailed unpaired Student’s *t* test. Scale bars: 500 μm.

**
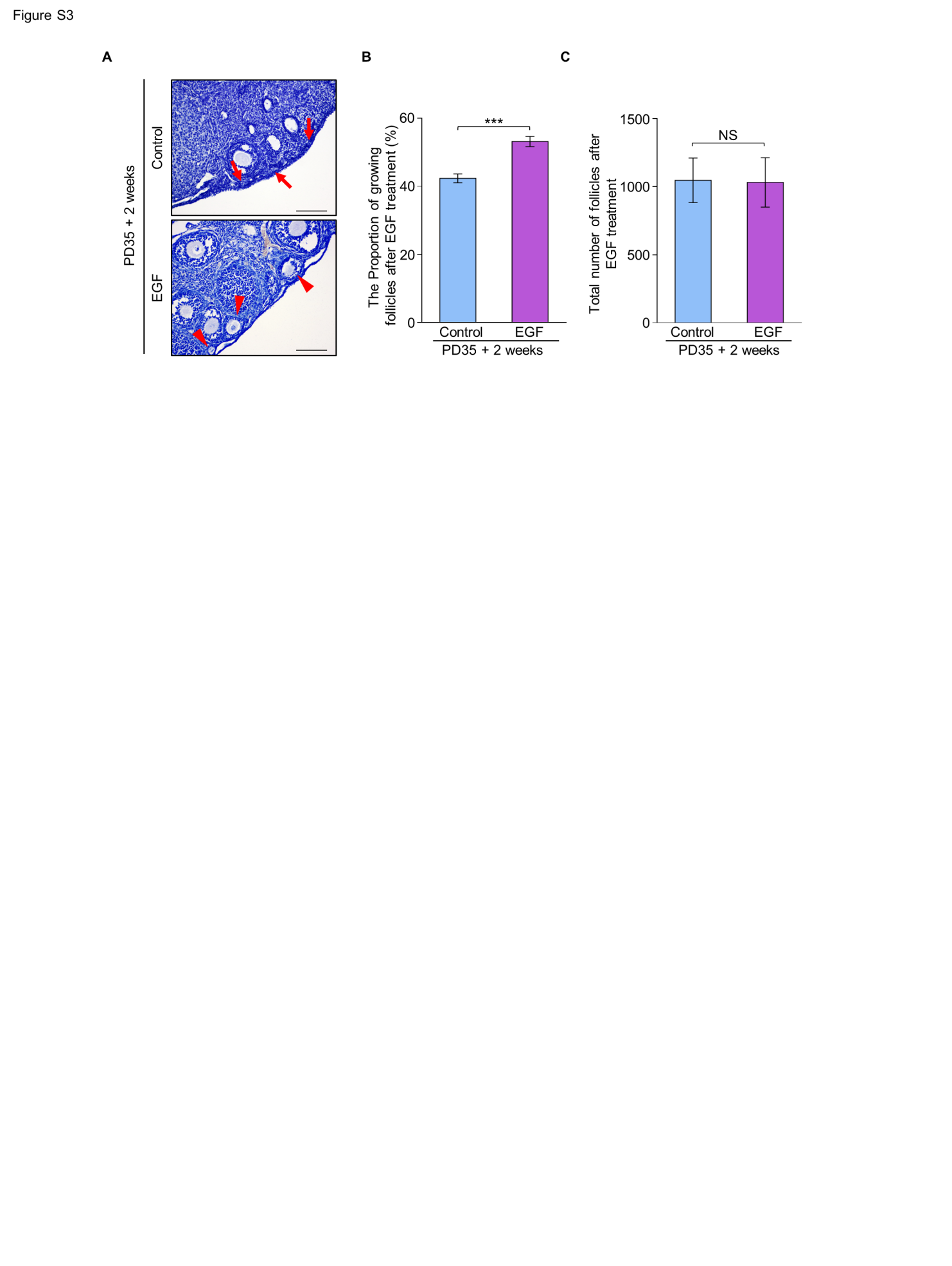
Figure S3**

**Figure S3. Ovarian topical administration of EGF with hyaluronan as a scaffold improved the activation of primordial follicles in the ovaries of adult mice.** (**A**) Two weeks after *in vivo* EGF treatment, histological analysis showed an increased number of growing follicles (arrowheads) in the cortical region of EGF-treated ovaries compared to the normal distribution of primordial follicles (arrows) in the control ovaries (n = 3). (**B**) Follicle counting revealed a significantly increased proportion of growing follicles in EGF-treated ovaries compared to the controls (53.17 ± 1.22% v.s. 42.34 ± 1.06%) (n = 3). (**C**) And an identical number of total follicles was observed in EGF-treated ovaries compared to that of the controls (1031.67 ± 147.98 v.s. 1047.33 ± 133.57) (n = 3). Data represent the mean ± SD of biological triplicate experiments. NS, *P* > 0.05 and ****P* < 0.001, by 2-tailed unpaired Student’s t test. Scale bars: 100 μm.

**
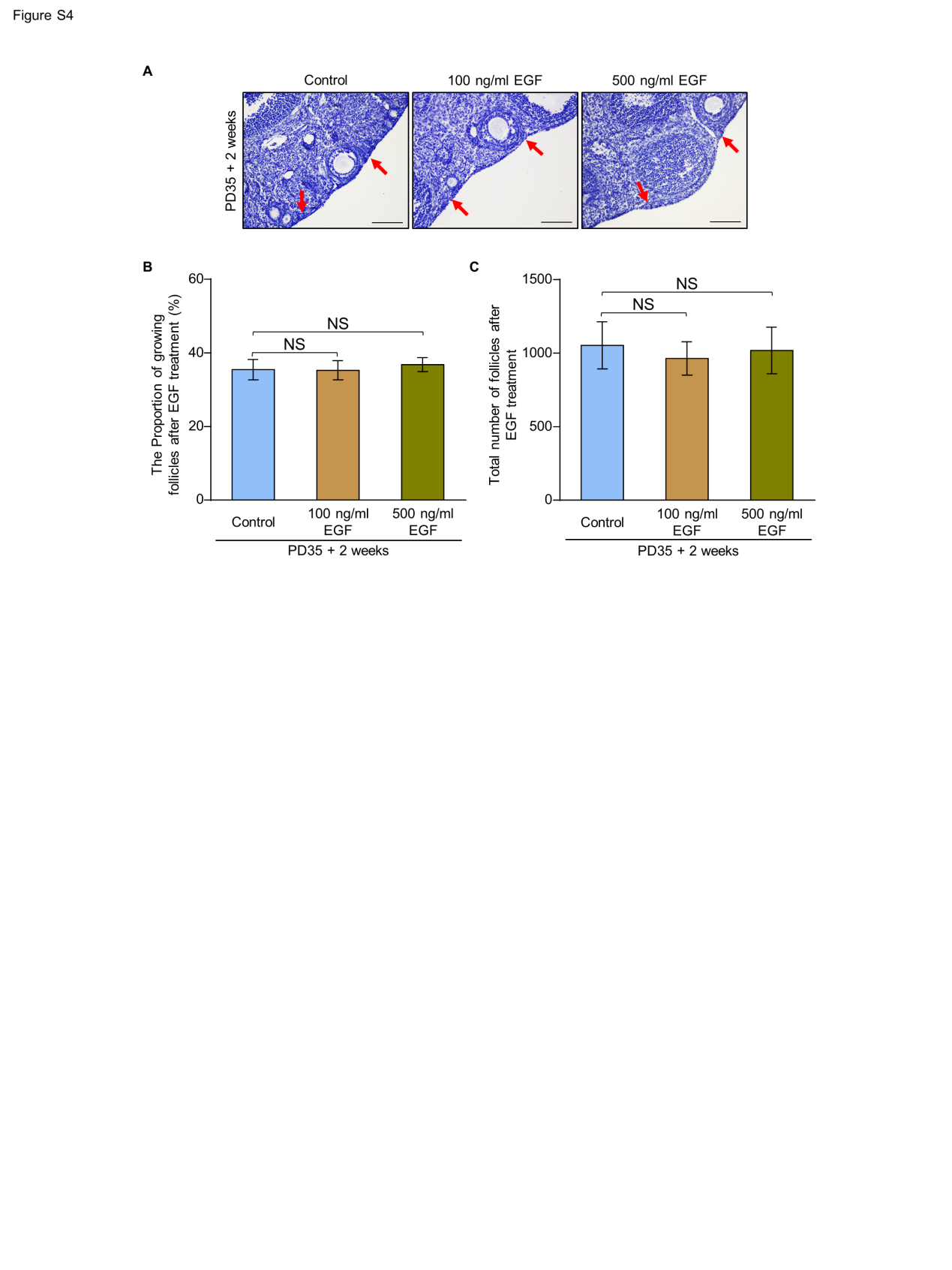
Figure S4**

**Fig. S4.** **Intraperitoneal injection of EGF had no effect on the activation of primordial follicles.** (**A**) Histological analysis showed an identical follicle distribution in intraperitoneal EGF treated ovaries and controls. (**B-C**) Follicle counting results showed no difference of both the proportion of growing follicles and the total number of follicles between intraperitoneal EGF treatment (n = 3) and control group (n = 3). NS, *P* > 0.05, by 2-tailed unpaired Student’s t test. Scale bars: 100 μm.

**Table S1**

| **Table S1. The number and distribution of follicles in fresh ovarian cortical pieces** | | | | | | |
| --- | --- | --- | --- | --- | --- | --- |
|  | primordial follicles | primary follicles | secondary follicles | antral follicles | total follicles | growing/total |
| patient 1 | 89 | 14 | 0 | 0 | 103 | 13.6% |
| patient 2 | 135 | 18 | 0 | 0 | 153 | 11.8% |
| patient 3 | 192 | 96 | 0 | 0 | 288 | 33.3% |
| patient 4 | 58 | 45 | 0 | 0 | 103 | 43.7% |
| patient 5 | 33 | 10 | 0 | 0 | 43 | 23.3% |

Human cortical ovarian pieces were collected from 5 patients who had PCOS. The number and distribution of follicles were detected in fresh ovarian cortical pieces before the transplantation.
